# CD3+T-lymphocyte infiltration is an independent prognostic factor for advanced nasopharyngeal carcinoma

**DOI:** 10.1186/s12885-020-06757-w

**Published:** 2020-03-21

**Authors:** Nasser Al-Rajhi, Hussein Soudy, Shoaib A. Ahmed, Tusneem Elhassan, Shamayel F. Mohammed, Hatim A. Khoja, Hazem Ghebeh

**Affiliations:** 1 Department of Radiation Oncology at King Faisal Specialist Hospital and Research Center, Riyadh, Saudi Arabia; 2 Department of Medical Oncology at King Faisal Specialist Hospital and Research Center, Riyadh, Saudi Arabia; 3 Clinical Oncology Department, Cairo University, Giza, Egypt; 4 St. George/Sutherland cancer Center, Sydney, Australia; 5 Leicester University Hospital UK, Leicester, UK; 6 Research Unit, Oncology Centre, King Faisal Specialist Hospital and Research Centre, Riyadh, Saudi Arabia; 7 Deparment of Laboratory Medicine and Pathology, King Faisal Specialist Hospital and Research Centre, Riyadh, Saudi Arabia; 8 Stem Cell & Tissue Re-engineering Program, Research Centre, King Faisal Specialist Hospital and Research Centre, PO Box 3354; Riyadh 11211; (MBC 03), Riyadh, Kingdom of Saudi Arabia

**Keywords:** Nasopharyngeal carcinoma, CD3, Immune infiltrate, Lymphocytes, Chemo-radiotherapy

## Abstract

**Background:**

Locally advanced nasopharyngeal carcinoma (LA-NPC) is a relatively rare disease in the west but more common in East Asia and areas of the Middle East like Saudi Arabia. Despite the advances in radiation therapy techniques, some patients relapse after treatment. In the coming era of cancer immunotherapy, prognostic factors for LA-NPC need to be further defined using immune-relevant markers. Several markers are available; however, the most robust and accessible/affordable marker is not well-defined.

**Methods:**

Retrospectively, tumor-infiltrating lymphocytes (TIL), their subsets as well as tumoral PD-L1 expression were analyzed in tumor tissues from 63 LA-NPC patients treated with platinum-based concurrent chemo-radiotherapy (CCRT) in addition to 20 cases with metastatic (MET) disease. Immunostaining was done using a validated and fully automated system. Scoring was done by two independent pathologists and results were compared.

**Results:**

There was no statistical difference between LA-NPC and MET disease in terms of CD3+, CD8+ TIL infiltration, or tumoral PD-L1 expression.

In LA-NPC, low CD3+ TIL infiltration highly correlated with shorter disease-free survival (DFS, HR = 8.5, *p* = < 0.001) and overall survival (OS, HR = 13, *p* = 0.015) with substantial agreement between scoring pathologists. A similar correlation was found between low CD8+ TIL and survival. Correlation of total TIL was significant with DFS (HR = 4.0, *p* = 0.008), borderline with OS and the correlation was dependent on the scoring pathologist. Having histological WHO type I&II correlated significantly with shorter DFS (HR 4.03, p = 0.008) and low CD3+ TIL (*p* = 0.009). Subgroup analysis of LA-NPC that included undifferentiated type (WHO type III) cases only (*n* = 58), showed a strong correlation between low CD3+ TIL and shorter DFS (HR = 7.2, *p* = < 0.001) and OS (HR = 17.3, *p* = 0.008). PD-L1 was expressed in 72% of type III LA-NPC cases while lacking PD-L1 expression correlated with shorter OS (HR = 6.1, *p* = 0.031). Patients with a combination of low CD3+ TIL and lack of PD-L1 expression had the worst OS (*p* < 0.001).

**Conclusions:**

CD3+ TIL is promising as a robust and independent prognostic marker for DFS and OS of LA-NPC patients treated with platinum-based CCRT. We would suggest the use of CD3 + TIL as a stratifying factor for LA-NPC, which warrants further validation in prospective trials.

## Background

Nasopharyngeal Carcinoma (NPC), a malignancy of the epithelial lining of the nasopharynx [1], is a distinct type of head and neck cancer. NPC has a strong association with Epstein-Barr virus (EBV) as a primary etiologic agent. Locally advanced nasopharyngeal carcinoma (LA-NPC), defined as NPC with stage III or IV_A,_ is common in Southern China, Southeast Asia, North Africa and some parts of the Middle East, including Saudi Arabia. Treatment of LA-NPC has been the main focus of NPC research in the past decades [2]. There remains a paucity of effective therapeutic options for this disease and hence, novel and effective therapy for NPC is urgently warranted [3, 4]. Importantly, accurate biomarkers that can predict the response to therapy are needed.

Platinum-based induction chemotherapy, followed by concurrent chemo-radiotherapy (CCRT), is the standard treatment approach for LA-NPC [2]. However, relapse after definitive treatment remains a potential challenge. Enthusiasm currently exists for an immunotherapeutic strategy that utilizes the anti-tumor ability of the immune system. Ongoing clinical trial (KEYNOTE-028) has established the clinical activity of the anti-PD-1 agent, pembrolizumab in recurrent/metastatic NPC [5]. A recent trial has shown that multiple immunotherapeutic agents can synergize to enhance endogenous antitumor immunity [6]. The subgroup of NPC patients who might be good responders to immunotherapy is yet to be defined. In addition, the best treatment strategy for LA-NPC is not well-identified at present.

The geographic variation in NPC distribution between different parts of the world and the promising role of immunotherapy for the treatment of NPC highlights the importance of studying the immune microenvironment in our local patients. We focused on LA disease, a commonly seen type of NPC patients in this part of the world and possibly other endemic areas.

In this study, we investigated the main components of the immune response elements as potential prognostic biomarkers to tailor the treatment strategies aiming at improving the outcome of LA-NPC. We have compared total TIL (using H&E sections) versus CD3+ or CD8+ TIL to predict the survival of LA-NPC patients. We have shown that low CD3+ TIL is a robust prognostic factor for shorter disease-free and overall survival of LA-NPC patients.

## Methods

### Patient selection

This study was conducted in accordance with institutionally approved guidelines and it was approved by the Research Advisory Council (RAC# 2150–013) of King Faisal Specialist Hospital and Research Centre (KFSH&RC). Retrospectively, 63 NPC patients with LA disease at presentation were reviewed and enrolled for this study. In addition, patients with MET disease at presentation (*n* = 20) were enrolled. In metastatic patients, the most common site for metastasis was bone (70%), followed by liver (25%), and lymph nodes (abdominal/pelvic) (25%), in addition to lung (15%).

All enrollments were among NPC patients seeking treatment at KFSH&RC between 2005 till 2016. All patients were initially evaluated at the combined head and neck clinic, where the diagnosis was confirmed by pathology and the complete blood count & differential (CBCD), renal and hepatic profile were done. Patients were staged using CT scan for head and neck, chest, abdomen and pelvis in addition to PET/CT scan and MRI for head and neck.

All patients were retrospectively staged according to the American Joint Committee on Cancer (AJCC 8th edition) 2018 staging system and they all were designated as stage III to IV_A_ for localized disease and thus considered LA-NPC. The MET cases were all labeled IV_B_ stage.

### Treatment

All LA-NPC patients (*n* = 63) received 2 cycles of induction chemotherapy (Epirubicin 70 mg/m^2^/Cisplatin 100 mg/m^2^) or (Docetaxel & Cisplatin 75 mg/m^2^ each) delivered at days 1 and 21. Patients were then treated with a definitive course of radiotherapy using IGRT helical (7000 cGy in 33 fractions over 6.5 weeks), starting on day 42, with two cycles of concurrent cisplatin 25 mg/m^2^ for 4 days on days 42 and 63. Metastatic patients were either treated with a combination of chemotherapy and radiation therapy (*n* = 7) or offered palliative care (*n* = 13). All patients were followed up at the head and neck clinic every 3 months for 3 years, 6 months for 2 years, then annually.

### Immunohistochemistry (IHC)

Formalin-fixed paraffin-embedded (FFPE) tissue blocks from tissue biopsy obtained at the time of diagnosis, before introducing any chemo or radiotherapy, were used for this study. Hematoxylin and eosin (H&E) sections were available from each tissue block as a routine hospital care for NPC patients.

Immunostaining was done on sections (4 μm) of the tissue blocks, which was mounted on glass slides and dried in an oven at 60 °C for 1 h. Immunohistochemistry was performed using a fully automated Ventana Benchmark Ultra (Ventana/Roche) system. The antigen retrieval was performed using the ULTRA CC1 solution (Ventana) and the immunostaining was done using Ventana’s validated reagents and ready-to-use primary antibodies (Supplementary Table 1) except FOXP3 antibody (clone 236A/E7, Abcam, USA). Antibody binding was detected using ultraView (all antibodies except PD-L1) or OptiView (for PD-L1) detection systems (both from Ventana). Permanent Red and 3,3′ diaminobenzidine (DAB) were used for signal visualization. For CD3/FOXP3 double-staining, CD3 staining with permanent Red was made first, followed by FOXP3 staining with DAB. A signal enhancer (from Ventana) was used for PD-1 staining.

### Pathological scoring

Two independent pathologists (HK & SM), who were blinded to the treatment outcome, scored and interpreted the histological tumor sections. Initially, total, CD3+, CD8+ TIL, FOXP3, and PD-1 scoring was done by one pathologist while PD-L1 scoring was done by the second pathologist. Due to a degree of subjectivity in TIL scoring, the second pathologist also scored total, CD3+ and CD8+ TIL independently for comparison. The initial assessment (primary pathologist) is described in the text and Tables 2, 3, 4, 5, and 6 and compared with the results of secondary pathologist scoring (described in the text only).

Scoring of lymphocyte infiltration, as adapted from Salgado et al. [7], was done in a semi-quantitative estimation giving 4-tier scale score. The score depends on the percentage of the total, CD3+ or CD8+ cells occupying the field. Score 1 was given for absent/rare TIL defined as TIL occupying < 10% of the filed. Similarly, scores of 2 (mild TIL), 3 (moderate TIL) and 4 (severe TIL) were given for TIL occupying 10–40%, 40–70 and > 70% of the field, respectively.

We have further dichotomized the scores into low and high (based on their average/median) for statistical analysis. In total (using H&E) and CD3+ TIL, both scores 1 and 2 were considered low TIL, while scores 3 and 4 were considered high TIL. Due to lower abundance of CD8+, the cutoff was decreased and therefore, score 1 was considered low TIL while scores 2, 3 and 4 were considered high TIL. Evaluation was done using × 20 objective and included general stromal TIL within the tumor area.

PD-L1 expression on tumor cells was scored 1–4, depending on the percentage of tumor cells expressing PD-L1 using the score/percentage cutoff used in TIL. Comparison was made between PD-L1 negative (< 10% of tumor cells express PD-L1 i.e. score 1) and PD-L1 positive (≥ 10% of tumor cells are positive, i.e. scores 2, 3 or 4).

For subsets CD3+ TIL, FOXP3 and PD-1 data were dichotomized using a 10% cutoff where FOXP3 expression in ≤10% of CD3+ TIL were considered low while PD-1+ cells in < 10% of CD3+ TIL were considered low.

### Statistical analysis

Student’s t-test (unpaired with equal variance) was used to compare statistical significance between LA and MET NPC cases. Levene’s test was used to check for equal variance. In the case of unequal variance, random samples were selected and the student’s t-test was applied again. Patient characteristics were summarized using frequencies and medians with ranges and compared using chi-square (χ2) for categorical variables. Survival probabilities were calculated using Kaplan-Meier methods and survival curves were compared using log-rank test. Overall survival (OS) was defined as time to death of any cause, while disease-free survival (DFS) was defined as time to relapse/progression or death. Patients who are alive and disease-free at last follow-up time was censored.

Associations between proposed risk factors and survival outcomes were evaluated using Cox (proportional hazard) regression (CR) models. All the variables were tested for the affirmation of the proportional hazard assumption and no variable violated the proportionality assumption. A stepwise model building was utilized to select the adjusted factors for each outcome with a threshold of 0.05 for both entry and stay in the model. Inter-observer variability was assessed using Cohen’s kappa coefficients to measure the level of agreement in scoring between pathologists. All analysis was performed using R studio and Prism 5, GraphPad, USA.

## Results

### Patient characteristics

Sixty-three patients with LA-NPC were included in this study with a median age of 45 years in addition to 20 cases of MET disease with a median age of 50 years (Table 1).

**Table 1:** Patients Characteristics

Age	LA (*n* = 63)	MET (n = 20)
	22–76 Median 45	16–78 Median 50
**Gender**		
Male	51 *(81)	14 (81)
Female	12 (19)	6 (19)
**WHO Type**		
I	1 (2)	0 (0)
II	4 (6)	3 (15)
III	58 (92)	17 (85)
**T stage**		
T1	19 (30)	2 (10)
T2	2 (3)	5 (25)
T3	16 (25)	4 (20)
T4	26 (41)	9 (45)
**N stage**		
N0	3 (5)	0 (0)
N1	10 (16)	1 (5)
N2	18 (29)	2 (10)
N3	32 (51)	17 (85)
**TNM Staging**		
III	14 (22)	0
IVa	49 (78)	0
IVb	0 (0)	20 (100)
**Relapse**		
No	42 (67)	NA
Yes	21 (33)	
**Type of Relapse**		
None	42 (67)	NA
Local	3 (5)	
Locoregional	2 (3)	
Systemic	16 (25)	
**Survival**		
Alive	53 (84)	7 (35)
Dead	10 (16)	13 (65)

*LA* Locally Advanced

*MET* Metastatic

*NA* Not applicable

* Percentage of cases

In agreement with the literature, the majority of patients were males. Importantly, in this cohort of patients, the majority were non-keratinizing undifferentiated carcinoma (WHO type III) and predominantly large tumors (T3 and T4) with high lymph node involvement (N) consistent with advanced NPC.

LA-NPC cases were followed up for a median time of 5 years from the time of diagnosis, of which 21 (31%) relapsed, including 5 patients with local or loco-regional and another 16 (25%) with systemic relapse. Of all the LA patients, eventually, 10 (16%) died of the disease.

### Immune cell infiltration, their subsets and PD-L1 expression in all NPC patients

**Fig. 1:**
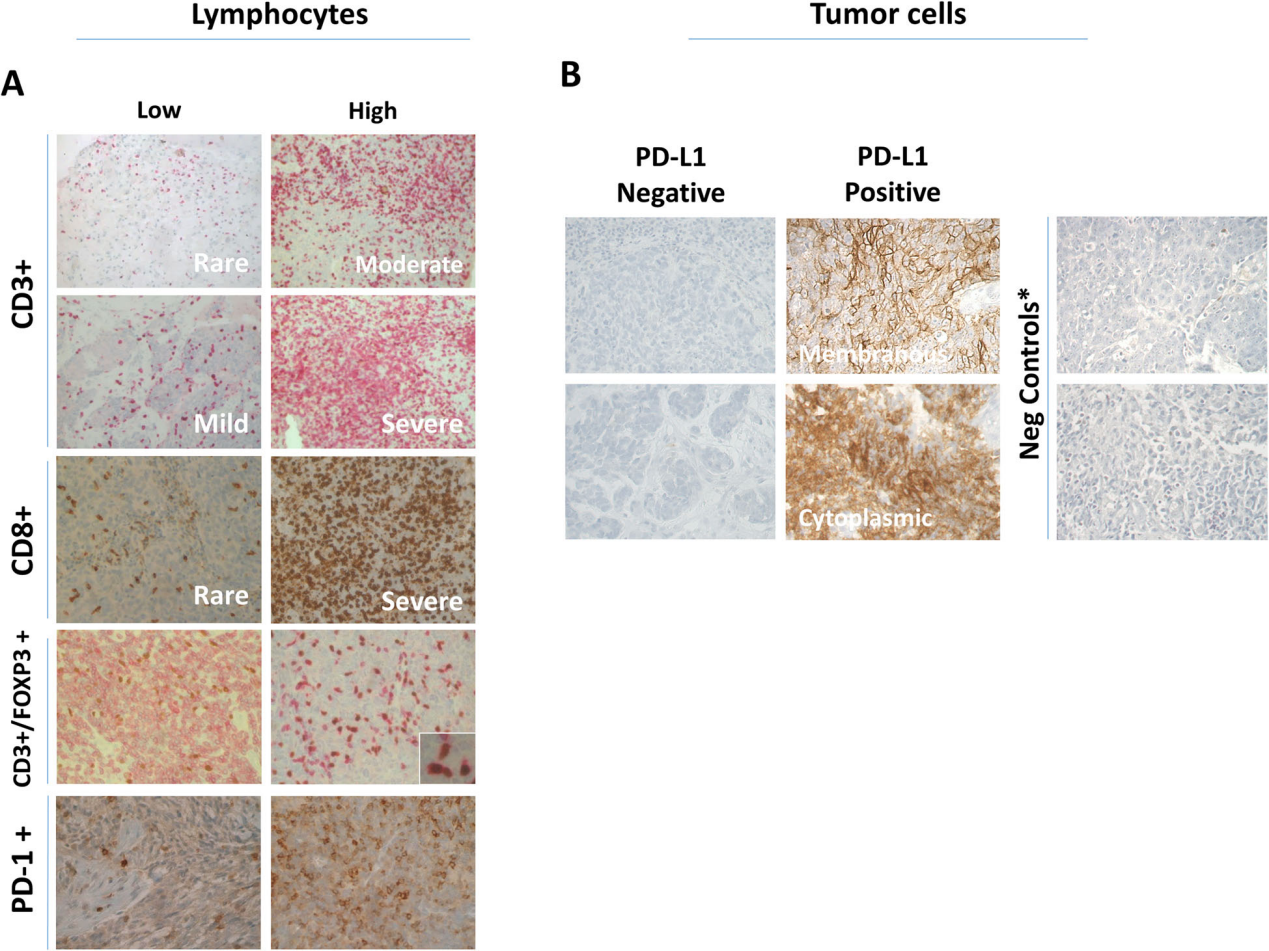
Immune cell infiltration and PD-L1 expression in nasopharyngeal carcinoma. Representative images at × 200 magnification of nasopharyngeal carcinoma tissue sections showing **a** T-lymphocytes and their subsets **b** Tumor cells expression of PD-L1. The red color represents CD3 staining while CD8, FOXP3 (nuclear), PD-1 and PD-L1 are brown. Inset in the FOXP3 staining image shows a higher magnification image (× 530) to illustrate the membranous red CD3 staining and nuclear brown FOXP3 staining as a typical marker for T-reg cells. *Images of negative controls are shown in **b**, i.e., sections incubated with antibody diluent alone without primary antibody

Total lymphocyte infiltration was assessed initially by simple H&E (total TIL) then immunostaining with CD3 or CD8. The infiltration score was dichotomized into low and high infiltration (see methods and materials) with each immune marker (Fig. 1 a). Scoring total TIL using H&E stained sections was more time consuming and straining for pathologists than scoring CD3 or CD8 stained sections.

PD-L1 expression on tumor cells of some patients was purely membranous while it was membranous and cytoplasmic (Fig. 1b) in other cases. The PD-L1 (whether membranous or cytoplasmic) was expressed in 70% of NPC cases.

Some of Immune biomarkers correlated with each other; for example, PD-L1 expression in tumor cells correlated significantly with CD3+ TIL (*p* = 0.015) (Table 2). CD3+ TIL highly correlated with total and CD8+ TIL (*p* < 0.001, data not shown) while there was no significant correlation between PD-L1 expression and FOXP3 + TIL or PD-1+ TIL (Table 2). On the other hand, some of the examined immune markers correlated with NPC standard prognostic markers (Table 3). For instance, a lack of PD-L1 expression correlated with advanced age and WHO type I&II (*p* = 0.009). Similarly, low CD3+ TIL infiltration correlated with WHO type I&II (*p* = 0.009) and higher T stage tumors (*p* = 0.036) (Table 3).

**Table 2:** Correlation of infiltration of TIL and expression of PD-L1 in tumor tissues of 83 NPC patients

	PD-L1 in Tumor cells		*P
	–	+	
**Total TIL**			
High	11 (24)^♣^	35 (76)	0.230
Low	14 (38)	23 (62)	
**CD3+ TIL**			
High	**9 (19)**	**39 (81)**	**0.015**
Low	**16 (46)**	**19 (54)**	
**CD8+ TIL**			
High	13 (23)	43 (77)	0.073
Low	12 (44)	15 (56)	
**FOXP3+/CD3+ TIL**			
Low (≤ 10% of CD3 + TIL)	15 (28)	38 (72)	
High (> 10% of CD3 + TIL)	10 (33)	20 (67)	0.629
^**⋄**^**PD-1 +/CD3+ TIL**			
Low (< 10% of CD3 + TIL)	9 (27)	24 (73)	
High (≥ 10% of CD3 + TIL)	16 (35)	30 (65)	0.625

(+ and -) are number of positive and negative patients, *P values in bold represent significant data as calculated using Fisher’s Exact Test, ♣Numbers between brackets are the percentages of patients, **⋄** Four samples had unknown PD-1 status

**Table 3:** Correlation of clinicopathological features with immunological markers in 83 patients with NPC (including LA-NPC and MET cases)

	CD3+ TIL		*P	CD8+ TIL		*P*	PD-L1 in Tumor Cells		*P*
	–	+		–	+		–	+	
**Age**									
< 40 years	7 (26)^♣^	20 (74)	0.057	5 (19)	22 (82)	0.080	**2 (7)**	**25 (93)**	**0.002**
≥ 40 years	28 (50)	28 (50)		22 (39)	34 (61)		**23 (41)**	**33 (59)**	
**WHO Type**									
I & II	**7 (88)**	**1 (12)**	**0.009**	5 (63)	3 (37)	0.106	**5 (63)**	**3 (37)**	**0.049**
III	**28 (37)**	**47 (63)**		22 (29)	53 (71)		**20 (27)**	**55 (73)**	
**T stage**									
1&2	**7 (25)**	**21 (75)**	**0.036**	6 (21)	22 (79)	0.298	5 (18)	23 (82)	0.127
3	**8 (40)**	**12 (60)**		8 (40)	12 (60)		9 (45)	11 (55)	
4	**20 (57)**	**15 (43)**		13 (37)	22 (63)		10 (31)	24 (69)	
**N stage**									
0&1	7 (50)	7 (50)	0.427	7 (50)	7 (50)	0.060	5 (36)	9 (64)	0.514
2	8 (40)	12 (60)		9 (45)	11 (55)		4 (20)	16 (80)	
3	20 (41)	29 (59)		11 (22)	38 (78)		16 (33)	33 (67)	
**M stage**									
0	27 (43)	36 (57)	1.000	19 (30)	44 (70)	0.424	18 (29)	45 (71)	0.587
1	8 (40)	12 (60)		8 (40)	12 (60)		7 (35)	13 (65)	
**UICC Stage**									
III	5 (36)	9 (64)	0.808	7 (50)	7 (50)	0.142	4 (29)	10 (71)	0.862
IVa	22 (45)	27 (55)		12 (24)	37 (76)		14 (29)	35 (71)	
IVb	8 (40)	12 (60)		8 (40)	12 (60)		7 (35)	13 (65)	

***Abbreviations***: *(+ and -)* are number of positive and negative patients, *P values in bold represent significant data as calculated using χ^2^ or Fisher’s Exact Tests, ^♣^Numbers between brackets are the percentages of patients

Altogether, low CD3+, CD8+ TIL and low PD-L1 correlated with each other and correlated with some of the known poor prognostic markers of NPC.

### Comparison of immune-related markers between LA and MET NPC cases

Initial analysis between immune biomarkers and metastasis (M stage) did not show any significant correlation (Table 3). As these data were dichotomized, we compared LA and MET cases using actual scores to check for any possible differences that were masked upon dichotomization. Again, there was no significant difference between CD3+ or CD8 infiltration in LA and MET patients (Supplementary Fig. 1a). There was no statistically significant difference in PD-L1 expression between LA and MET cases, as well. Similarly, there was no significant difference between FOXP3+ or PD-1+ subsets of CD3+ T-cells between LA and MET cases (Supplementary Fig. 1a).

Among LA-NPC patients who relapsed and developed metastasis, we had tissue blocks for biopsies from metastatic sites of 6 patients. To further investigate differences between LA and MET cases, we compared immune biomarkers in primary tumors before metastasis with metastatic sites after developing metastasis in these 6 paired cases. Even with paired cases, there was no statistically significant difference between LA and MET cases in terms of the degree of CD3+ or CD8+ TIL infiltration or PD-L1 expression (Supplementary Fig. 1b). However, there was a consistent and significant decrease (*p* = 0.033) in the percentage of FOXP+/CD3+ (T-reg) cells in the metastatic sites compared with the primary tumor. Altogether, there was no statistically significant difference in TIL, their subsets, or tumoral PD-L1 between LA and MET cases.

### Correlation of immune-related markers and disease outcome in LA-NPC patients

We then looked specifically at LA-NPC cases to correlate the immune-related prognostic factors with disease outcome.

Due to the nature of the TIL assessment, which is characterized by some degree of subjectivity, we asked both pathologists involved to score total, CD3+ and CD8+ TIL. There was a fair degree of agreement between pathologists in relation to total TIL and CD8+ TIL (Cohen’s κ 0.58 and 0.57 for total and CD8+ TIL respectively) and substantial agreement in CD3+ TIL scoring (Cohen’s κ 0.61) (Supplementary Table 2). We then analyzed the scoring data of each pathologist independently to mimic real-world scenario.

**Fig. 2:**
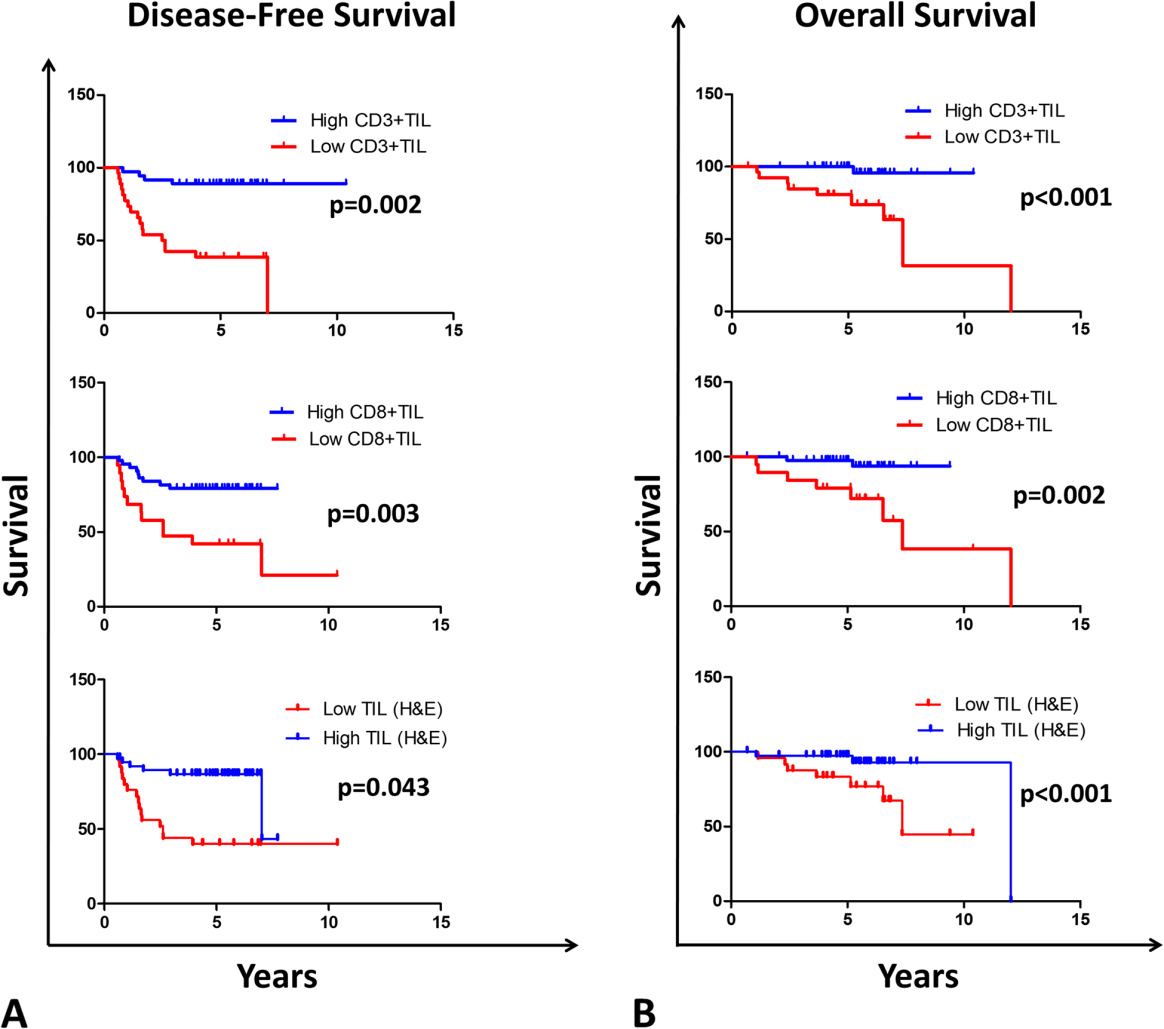
Relation of total, CD3+ and CD8+ TIL to survival of LA-NPC patients (*n* = 63). Kaplan–Meier survival curves showing **a** disease-free survival (DFS) and **b** overall survival (OS) of patients in relation to their CD3+ TIL infiltration, CD8+ TIL infiltration or total TIL (assessed from H&E sections) infiltration. Statistical significance was calculated using log-rank test

Kaplan-Meier survival curves showed a statistically significant separation between the low and high CD3+ TIL groups of patients (Fig. 2 a, log-rank *p* = 0.002). Univariate Cox regression (CR) model analysis showed a highly significant association between low CD3+ TIL and shorter disease-free survival (DFS) whether scoring was done by the primary (CR, HR 8.5, *p* = < 0.001) (Table 4) or the secondary pathologist (CR, HR 5.8, *p* < 0.001) (data not shown).

**Table 4:** Univariate Cox proportional hazard regression analysis of clinicopathological features and immunological markers with disease-free survival (DFS) and Overall Survival (OS) in 63 patients with LA-NPC at the time of presentation

	Relapse			DFS			Death			OS		
	–	+		HR	95% CI	*P	–	+		HR	95% CI	*P
**Age**												
40 years	17 (74)^♣^	6 (26)		1			21 (91)	2 (9)		1		
≥ 40 years	25 (62)	15 (38)		1.47	0.57–3.8	0.424	32 (80)	8 (20)		2.0	0.42–9.70	0.383
**WHO Type**												
III	**42 (72)**	**16 (28)**		**1**			50 (86)	8 (14)		1		
I & II	**0 (0)**	**5 (100)**		**4.0**	**1.42–11.4**	**0.008**	3 (60)	2 (40)		1.0	0.12–8.1	0.999
**T stage**												
1&2	18 (86)	3 (14)		1		0.125	21 (100)	0 (0)		1		0.988
3	9 (56)	7 (44)	1&2 vs 3	3.7	1.0–14.4	0.058	13 (81)	3 (19)	1&2 vs 3	133,198	0.0–6.45E+ 140	0.941
4	15 (58)	11 (42)	1&2 vs 4	3.5	1.1–12.5	0.057	19 (73)	7 (27)	1&2 vs 4	120,743	0.0–5.84E+ 140	0.941
**N stage**												
0&1	9 (69)	4 (31)		1		0.698	11 (85)	2 (15)		1		0.372
2	10 (56)	8 (44)	0&1 vs 2	1.5	0.45–5.0	0.501	13 (72)	5 (28)	0&1 vs 2	3.1	0.36–26.7	0.307
3	23 (72)	9 (28)	0&1 vs 3	1.0	0.32–3.4	0.947	29 (91)	3 (9)	0&1 vs 3	1.3	0.13–12.2	0.843
**UICC Stage**												
III	9 (64)	5 (36)		1			11 (79)	3 (21)		1		
IVa	33 (67)	16 (33)		0.9	0.37–2.9	0.898	42 (86)	7 (14)		0.5	0.12–2.0	0.328
**Total TIL**												
High	**29 (83)**	**6 (17)**		**1**			33 (94)	2 (6)		1		
Low	**13 (46)**	**15 (54)**		**4.0**	**1.2–10.7**	**0.008**	20 (71)	8 (29)		4.0	0.82–19.2	0.087
**CD3+ TIL**												
High	**32 (89)**	**4 (11)**		**1**			**35 (97)**	**1 (3)**		**1**		
Low	**10 (37)**	**17 (63)**		**8.5**	**3.1–29.6**	**< 0.001**	**18 (67)**	**9 (33)**		**13.1**	**1.64–100**	**0.015**
**CD8+ TIL**												
High	**35 (80)**	**9 (20)**		**1**			**42 (95)**	**2 (5)**		**1**		
Low	**7 (37)**	**12 (63)**		**3.7**	**1.5–8.8**	**0.004**	**11 (58)**	**8 (42)**		**7.6**	**1.6–37.0**	**0.011**
**FOXP3+/CD3+**												
Low (≤ 10% of CD3 + TIL)	30 (73)	11 (27)		1			37 (90)	4 (10)		1		
High (> 10% of CD3 + TIL)	12 (55)	10 (46)		1.6	0.7–3.8	0.287	16 (73)	6 (27)		1.8	0.5–7.5	0.376
^**⋄**^**PD-1 +/CD3+ TIL**												
Low (< 10% of CD3 + TIL)	16 (62)	10 (38)		1			22 (85)	4 (15)		1		
High (≥ 10% of CD3 + TIL)	24 (71)	10 (29)		0.6	0.2–1.5	0.260	29 (85)	6 (15)		0.60	0.2–1.5	0.260
**PD-L1 in Tumor (mem or cyto)║**												
Positive (≥ 10%)	32 (71)	13 (29)		1			12 (67)	6 (33)		1		
Negative (< 10%)	10 (56)	8 (44)		1.6	0.7–3.8	0.315	41 (91)	4 (9)		3.1	0.8–11.49	0.100
**PD-L1 in Tumor (cyto)**												
Positive (≥ 10%)	22 (69)	10 (31)		1			25 (81)	6 (19)		1		
Negative (< 10%)	20 (65)	11 (35)		1.1	0.4–2.5	0.919	28 (88)	4 (12)		1.3	0.34–4.9	0.702

*Abbreviations*: *(+ and -)* are number of positive and negative patients, *P values in bold represent significant data, ^♣^Numbers between brackets are the percentages of patients, **⋄**Three samples had unknown PD-1 status. **║** mem membranous**,** cyto cytoplasmic

Similarly, Kaplan-Meier survival curves showed a statistically significant separation between the low and high CD8+ TIL groups of patients (log-rank *p* = 0.003). In agreement, univariate CR model showed low CD8+ TIL, scored by the primary pathologist, to correlate significantly with DFS (CR, HR 3.7, *p* = 0.004) (Table 4) while the significance was borderline when scoring was done by the secondary pathologist (CR, HR 2.4, *p* = 0.055) (data not shown). While low and high total TIL survival curves were significantly separated (log-rank *p* = 0.043), low total TIL correlated significantly with shorter DFS only when scoring was done by the primary pathologist (CR, HR 4.0, *p* = 0.008) and not by the secondary pathologist (data not shown).

Low CD3+ TIL correlated significantly with shorter overall survival (OS) whether scored by the primary pathologist (CR, HR 13.1, *p* = 0.015) (Table 4) or the secondary pathologist (CR, HR 5.0, *p* = 0.045) (data not shown). In agreement, Kaplan-Meier curves were significantly separated (Fig. 2b, log-rank *p* < 0.001). Similarly, low CD8+ TIL correlated significantly with shorter OS when scoring was done by the primary pathologist (CR, HR 7.6, *p* = 0.011) while the significance was borderline when scoring was done by the secondary pathologist (CR, HR 3.4 *p* = 0.072). In agreement, low and high CD8+ TIL were significantly separated in Kaplan-Meier curves (log-rank *p* = 0.002). On the other hand, while low and high total TIL significantly separated (log-rank *P* < 0.001), the total TIL, irrespective of the scoring pathologist, did not correlate significantly with OS.

In addition to TIL, there was a statistically significant association between NPC tumors with WHO histological type I&II and shorter DFS (CR, HR = 4, *p* = 0.008) (Table 4). On the other hand, subsets of CD3+ TIL like FOXP3+ and PD-1+ TIL did not correlate significantly with DFS or OS (Table 4). Similarly, there was no significant correlation of tumoral PD-L1 with DFS or OS.

In multivariate CR analysis, only correlation of low CD3+ TIL with DFS or OS remained significant (*p* < 0.001, Table 5), while low PD-L1 showed borderline significance with OS.

**Table 5:** Multivariate Cox proportional hazard analysis of clinicopathological features and immunological markers with disease-free survival (DFS) and overall survival (OS) in 63 patients with LA-NPC at the time of presentation

	DFS			OS		
	HR	95% CI	*P	HR	95% CI	*P*
Age						
< 40 years	1			1		
≥ 40 years	0.7	0.2–2.6	0.624	1.3	0.1–32.3	0.839
WHO type						
III	1			1		
I & II	1.5	0.4–4.8	0.516	0.2	0.0–1.3	0.094
UICC Stage						
III	1					
IVa	1.4	0.4–4.8	0.597	0.6	0.1–5.6	0.604
**CD3+ TIL**						
High	**1**			**1**		
Low	**8**	**2.5–30.6**	**< 0.001**	**17.5**	**2.1–434.0**	**0.006**
FOXP3+/CD3+ TIL						
Low (≤ 10% of CD3 + TIL)	1			1		
High (> 10% of CD3 + TIL)	1.1	0.4–2.8	0.889	1.8	0.3–18.7	0.564
^**⋄**^ PD-1+/CD3+ TIL						
Low (< 10% of CD3 + TIL)	1					
High (≥ 10% of CD3 + TIL)	0.6	0.2–1.7	0.331	0.2	0–1.5	0.117
PD-L1						
Positive (≥ 10%)	1					
Negative (< 10%)	1.3	0.4–3.8	0.672	5.6	1–49.6	0.054

*P values in bold represent significant data. **⋄**Three samples had unknown PD-1 status. **║** mem = membranous**,** cyto = cytoplasmic

Altogether, densities of total, CD3+ and CD8+ TIL could significantly separate patient’s survival using Kaplan-Meier’s curves. In univariate and multivariate CR model analysis, only low CD3+ TIL correlated significantly with DFS and OS, supporting for CD3 + TIL as an independent prognostic factor for disease outcome of LA-NPC.

### Subgroup analysis of undifferentiated LA-NPC (WHO type III) patients with disease outcome

WHO type I&II correlated with shorter DFS but not OS. However, the majority of the patients were WHO type III patients. Therefore, we did a subgroup analysis of WHO type III patients only.

**Fig. 3:**
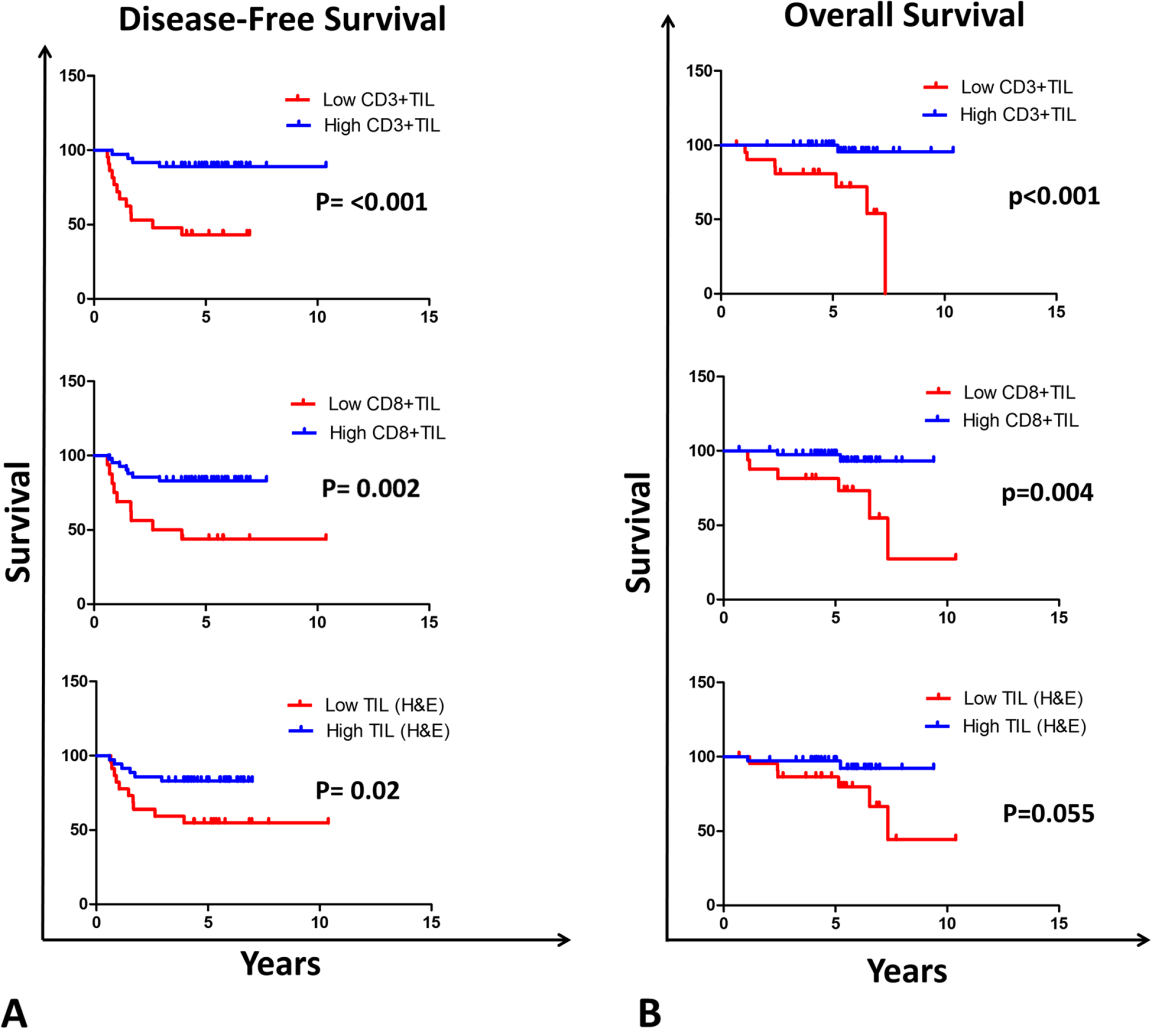
Relation of total, CD3+ and CD8+ TIL to survival of subgroup of LA-NPC patients with undifferentiated histological WHO type III (*n* = 58). Kaplan–Meier survival curves showing **a** disease-free survival (DFS) and **b** overall survival (OS) of patients in relation to their CD3+ TIL infiltration, CD8+ TIL infiltration or total TIL (assessed from H&E sections) infiltration. Statistical significance was calculated using log-rank test

In histological WHO type III patients, the univariate CR analysis model showed a highly significant association of low total TIL (HR 3.1, *p* = 0.029), low CD3+ TIL (HR 7.2, *p* < 0.001) and low CD8+ TIL (HR 4.3, *p* = 0.004) with shorter DFS (Table 6). Similarly, low CD3+ and CD8+ TIL correlated with OS (HR 17.3, *p* = 0.008 and HR 7.5, 0.014 respectively), while the association between total TIL and OS was borderline. Likewise, separation of Kaplan-Meier survival curves based on TIL densities was statistically significant for shorter DFS and OS with low CD3+ or CD8+ TIL in type III LA-NPC patients (Fig. 3). A similar association was seen for low total TIL, although it was significant with shorter DFS and borderline with shorter OS.

**Table 6:** Univariate Cox proportional hazard regression analysis of clinicopathological features and immunological markers with disease-free survival (DFS) or overall survival (OS) in subgroup of undifferentiated WHO type III LA-NPC patients (*n* = 58)

	Relapse		DFS			Death		OS		
	–	+	HR	*(95% CI)*	*P	–	+	HR	*(95% CI)*	*P*
**Total TIL**										
High	**29 (83)**^♣^	**6 (17)**	**1**			33 (94)	2 (6)	1		
Low	**13 (56)**	**10 (44)**	**3.1**	**1.1–9.1**	**0.029**	17 (74)	6 (26)	4.3	1–29.6	0.075
**CD3+ TIL**										
High	**32 (89)**	**4 (11)**	**1**			**35 (97)**	**1 (3)**	**1**		
Low	**10 (45)**	**12 (55)**	**7.2**	**2.5–25.7**	**< 0.001**	**15 (68)**	**7 (32)**	**17.3**	**3.0–324.4**	**0.008**
**CD8+ TIL**										
High	**35 (83)**	**7 (17)**	**1**			**40 (95)**	**2 (5)**	**1**		
Low	**7 (44)**	**9 (56)**	**4.3**	**1.6–11.9**	**0.004**	**10 (62)**	**6 (38)**	**7.5**	**1.7–51.3**	**0.014**
**PD-L1+ tumor cells (mem or cyto)║**										
Positive (≥ 10%)	32 (76)	10 (24)	1			**39 (93)**	**3 (7)**	**1**		
Negative (< 10%)	10 (63)	6 (37)	1.7	0.6–4.5	0.313	**11 (69)**	**5 (31)**	**6.1**	**1.3–42.7**	**0.031**

*Abbreviations*: *(+ and -)* are number of positive and negative patients, ^♣^Numbers between brackets are the percentages of patients, *P values in bold represent significant data. **║** mem = membranous**,** cyto = cytoplasmic

Interestingly, in WHO type III patients, low PD-L1 expression correlated significantly with shorter OS (HR 6.1, *p* = 0.031) and Kaplan-Meier survival curves could be significantly segregated based on PD-L1 expression (log-rank *p* = 0.067, Supplementary Fig. 2). In multivariate CR analysis, the correlation of low CD3+ TIL infiltration with DFS and OS remained significant (Supplementary Table 3). Similarly, the correlation of PD-L1 with OS remained significant in multivariate CR analysis (*p* < 0.001). In addition to PD-L1, PD-1 expression became significant in multivariate analysis; however, the significance was unstable as it was dependent on the presence of PD-L1 as a variable. In addition, Kaplan-Meier survival curve for PD-1 expression was not significant (data not shown).

Altogether, in WHO type III patients, low CD3+ TIL correlated significantly with shorter DFS and OS, as shown in univariate and multivariate CR analysis. Low PD-L1 expression correlated with OS in both univariate and multivariate CR analyses.

### Combination of PD-L1 expression and CD3+ or CD8+ TIL infiltration in WHO type III patients

As both CD3+ TIL and tumoral PD-L1 contributed to OS in WHO type III patients, we used a combination analysis of CD3+ TIL infiltration and PD-L1 expression in tumor cells. Results show that patients having tumors that are PD-L1 negative and lack CD3+ TIL (Type II) are associated with the shortest OS among other combinations while patients with tumors that are positive for PD-L1 expression and CD3+ TIL (Type I) have the longest OS (Supplementary Table 4). In agreement, Kaplan-Meier survival curves segregated patients into the four types of CD3 & PD-L1 combinations (log-rank *p* < 0.001). Similar findings were seen with combination analysis of CD8+ TIL infiltration and tumor cells expression of PD-L1 (data not shown).

Altogether, in the subgroup of WHO type III NPC tumors, the combination of low CD3+ TIL and lack of PD-L1 expression correlated with shorter OS.

## Discussion

Despite the recent advances in radiation therapy techniques, which have improved the treatment outcome, a considerable fraction of patients with locally advanced nasopharyngeal carcinoma (LA-NPC) will eventually relapse. It is of utmost importance to identify robust prognostic factors to estimate the chances of relapse of LA-NPC as patients have shorter median survival once being diagnosed with recurrent and/or MET disease. Our study is the first to be dedicated to LA-NPC and shows that CD3+ TIL is an independent and robust prognostic factor for LA-NPC treated with standard CCRT. While high CD3+ TIL patients generally did well, many of the low CD3+ TIL relapsed and some succumbed to their disease.

NPC is known as “lymphoepithelioma” due to heavy infiltration with lymphocytes; nevertheless, significant variations still exist in the level of lymphocyte infiltration and, more importantly, as we have shown in this report the type of lymphocytes infiltrating the tumor. Assessment of TIL by pathologists has a degree of subjectivity. The use of digital image analysis has started to be used in pathology [8]. However, its application is still limited due to lack of standardization, validation availability and/or high cost. Assessment of tumor sections by pathologists still remains the backbone of any histological tumor evaluation. We used a previously defined and published pathologist-based scoring method [7]. We compared for the first time, as far as we know, between total, CD3+ and CD8+ TIL scoring in LA-NPC. We have shown in this report that while the correlation of total TIL using H&E sections with disease outcome was dependant on the scoring pathologist, the correlation CD3+, and to a lesser extent CD8+, with disease outcome was consistent and more robust. Indeed, the agreement in scoring between both pathologists was highest using CD3+ TIL (Cohen ‘s κ of 0.61) as compared for κ coefficient of 0.58 and 0.57 for total and CD8+ TIL, respectively (Supplementary Fig. 2).

Several studies have shown the prognostic value of TIL in various types of solid tumors [9], including Head and Neck cancers [10], and more specifically, NPC [11–14], although their studies were not devoted to LA-NPC. Wang et al. [13] and Almangush et al. [15] have studied total TIL in NPC using H&E stained sections and found a high correlation between total TIL and disease outcome using a large cohort (1490 patients). On the other hand, Zhu et al. [14], Lu et al. [11], and Ono et al. [12] investigated specific TIL like CD3+ and/or CD8+ TIL on survival of NPC. However, conclusions from these last three studies, in relation to CD3 + TIL, are not similar, possibly due to differences in methodology. The first two studies used computer-based image analysis software that counted cells and used the median as the cutoff. In addition, Zhu et al. [14] analyzed stromal (peritumoral) CD3 as a percentage rather than density i.e., an increase in NK or B cells would decrease the CD3 ratio. Therefore, all these details might affect the final conclusion about the most robust, reproducible and available method to evaluate TIL in NPC. We have used a well-described, straightforward method previously published by Salgado et al. [7] that depends on percentage of field occupied by TIL rather than their absolute number. We have evaluated and compared, for the first time, total TIL (using H&E sections) versus CD3+ or CD8+ TIL (requires immunostaining) to predict survival of LA-NPC patients. Our study showed that CD3+ TIL is robust, and to a lesser degree CD8+ TL, while total TIL evaluation (using H&E staining) is more dependent on the scoring pathologist.

We compared for the first time, the immune mapping between LA and MET disease in NPC. Our data did not show a significant or consistent difference in the expression of PD-L1, total, CD3+, or CD8+ TIL and/or their subsets between LA and MET disease. However, there were lower FOXP3+ regulatory T-cells in metastatic sites than the primary tumor. The significance of this finding is not clear.

As expected, we found very high correlation (*p* < 0.001) between CD3+ and CD8+ TIL as usually CD3+ TIL are composed of both CD4+ and CD8+ TIL. In principle, CD8+ TIL requires cytokines release by CD4+ TIL, IL-2 for example, for their proliferation [16]. Therefore, it is likely that the CD8+ TIL association with a good prognosis is due to general CD3+ T-lymphocyte infiltration.

Our data show a positive expression of PD-L1 in 70% of cases, which is consistent with previous reports [12, 17, 18]. The correlation between PD-L1 expression and better prognosis is consistent with Zhu et al. [14] and Lee et al. [18] who found PD-L1 to correlate with favorable prognosis in NPC. Interestingly, when CD3+ TIL was combined with PD-L1 expression status as done previously for CD8+ TIL and PD-L1 by Ono et al. [12], there was a correlation between patients having combination of low CD3 + TIL and lack tumoral PD-L1 (type II) with shorter OS.

It is becoming increasingly evident that antitumor immune response plays a major role in the eradication and/or control of many types of cancer, including head and neck tumors. Indeed, anti-PD-1 inhibitors are now approved as a second line for the treatment of recurrent or metastatic head and neck squamous cell carcinoma (RM-HNSCC) [19]. Despite the initial signs of efficacy of immunotherapy in head and neck cancers in general, it’s quite evident that the response to immune checkpoint inhibitors in several types of cancer usually correlates with pre-existing immune response (hot tumors). Based on our study and others, high TIL patients had a higher chance of survival in NPC; however, it is unkonwn whether immunotherapy, alone or in combination with other modalities, would improve the outcome of low TIL of LA-NPC patients. Further investigations and different treatment strategies should be tested in low and high TIL LA-NPC patients.

An important advantage of this study is that the treatment was consistent for all patients with induction chemotherapy consisting of cisplatin with either docetaxel or epirubicin followed by cisplatin-based concurrent chemo-radiotherapy. Different strategies can be attempted in the future to convert Low CD3 + TIL LA-NPC (possibly cold tumors) into hot tumors in order to improve LA-NPC treatment outcome. These strategies (reviewed in Bonaventura et al. [20]) include epigenetic drugs like demethylating agents to reverse silencing of TH1-type chemokines, tumor vaccines and oncolytic viruses to improve the immunogenicity of tumors, antiangiogenic and anti-TGF-β therapies to reverse exclusion of immune cells from tumor bed and immune cytokines to promote lymphocytes expansion at the tumor site.

One of the limitations of this study is the small number of patients. However, even with this small number of patients, we found CD3+ TIL as the strongest prognostic and/or predictive factor. Future studies will be planned for a much larger number of patients in prospective settings to validate our findings. On the other hand, all staining in this study was done using a fully automated and validated system.

Currently, there is no standard validated method for assessing TIL infiltrating in cancer tissues. We have used guidelines highlighted by Salgado et al. [7] as a practical method for pathologist-based scoring. However, other methods could also resolve this issue, including digital scoring [8]. The latter method has advantages of spatial analysis in addition to TIL phenotyping. In addition, machine learning could be integrated to resolve complex immune cell interactions [21]. However, more work still needs to be done to standardize and validate the staining and analysis methodology.

Altogether, CD3+ TIL is a potentially robust and independent prognostic factor for LA-NPC patients treated with standard CCRT.

## Conclusion

In NPC, there was no significant difference between LA and MET disease with regard to the immune mapping investigated in this study. Low CD3+ TIL in LA-NPC correlated significantly with shorter DFS (HR = 8.5, *p* = < 0.001) and OS (HR = 13.1, *p* = 0.015). Lack of PD-L1 correlated with shorter OS (HR 6.1, *P* = 0.031) in a subgroup of patients with WHO type III LA-NPC tumors. We have demonstrated that CD3+ TIL is an independent and robust prognostic marker for LA-NPC treated with standard CCRT. We would suggest the use of CD3 + TIL as a stratifying factor for LA-NPC, which warrants further validation in prospective trials.

## Supplementary information

**Additional file 1: Supplementary Figure 1. Expression of immune-related markers in LA and MET nasopharyngeal tumors.** Difference in CD3+ TIL, CD8+ TIL infiltration, subsets of TIL infiltration and Tumoral PD-L1 expression (membranous or cytoplasmic) between (A) locally advanced (LA, *n* = 63) versus metastatic (MET, *n* = 20) nasopharyngeal carcinoma cases or (B) primary tumor (1ry) and metastatic sites (MT sites) in available paired tissues from some of the LA patients before and after relapse and development of metastasis (n = 6). * Numbers indicate the median.

**Additional file 2: Supplementary Figure 2. Relation of tumoral PD-L1 expression to survival of WHO type III LA-NPC patients (*n*** **= 58).** Kaplan–Meier survival curves showing overall survival (OS) of WHO type III LA-NPC patients, in relation to their tumoral PD-L1 expression. Statistical significance was calculated using log-rank test.

**Additional file 3: Supplementary Figure 3. Correlation of microenvironment type with survival of LA-NPC patients.** Kaplan–Meier survival curves showing OS of WHO type III LA-NPC patients (n = 58) in relation to their microenvironment type based on CD3+ TIL and PD-L1 expression. Type I: Tumoral PD-L1 is positive and CD3 TIL is high, Type II: PD-L1 is negative while CD3 is low, Type III: tumoral PD-L1 is positive while CD3 TIL is low and Type IV: PD-L1 is negative while CD3 TIL is high. Statistical significance was calculated using log-rank test.

**Additional file 4: Supplementary Table 1.** List of Ventana antibodies used in the study.

**Additional file 5: Supplementary Table 2.** Agreement between the two scoring pathologists.

**Additional file 6: Supplementary Table 3** Multivariate Cox proportional hazard analysis of clinicopathological features and immunological markers with disease-free survival (DFS) and overall survival (OS) in 58 patients with WHO type III LA-NPC at the time of presentation.

**Additional file 7: Supplementary Table 4.** Univariate Cox proportional hazard regression analysis of different CD3 + TIL/Tumor PD-L1 expression combination types with overall survival (OS) in 58 patients with local NPC at time of presentation.

## Data Availability

The datasets generated during and/or analyzed during the current study are included in this published article (and its supplementary information files), otherwise available from the corresponding author on reasonable request.

## Abbreviations

NPC: Nasopharyngeal Carcinoma
CCRT: Concurrent chemo-radiotherapy
LA: Locally advanced
MET: Metastatic
OS: Overall Survival
DFS: Disease-free survival
