# Supplementary figures and images for: CD3+T-lymphocyte infiltration is an independent prognostic factor for advanced nasopharyngeal carcinoma
Source: BMC Cancer. 2020 Mar 21;20:240. doi: 10.1186/s12885-020-06757-w (PMC7227256; doi:10.1186/s12885-020-06757-w)

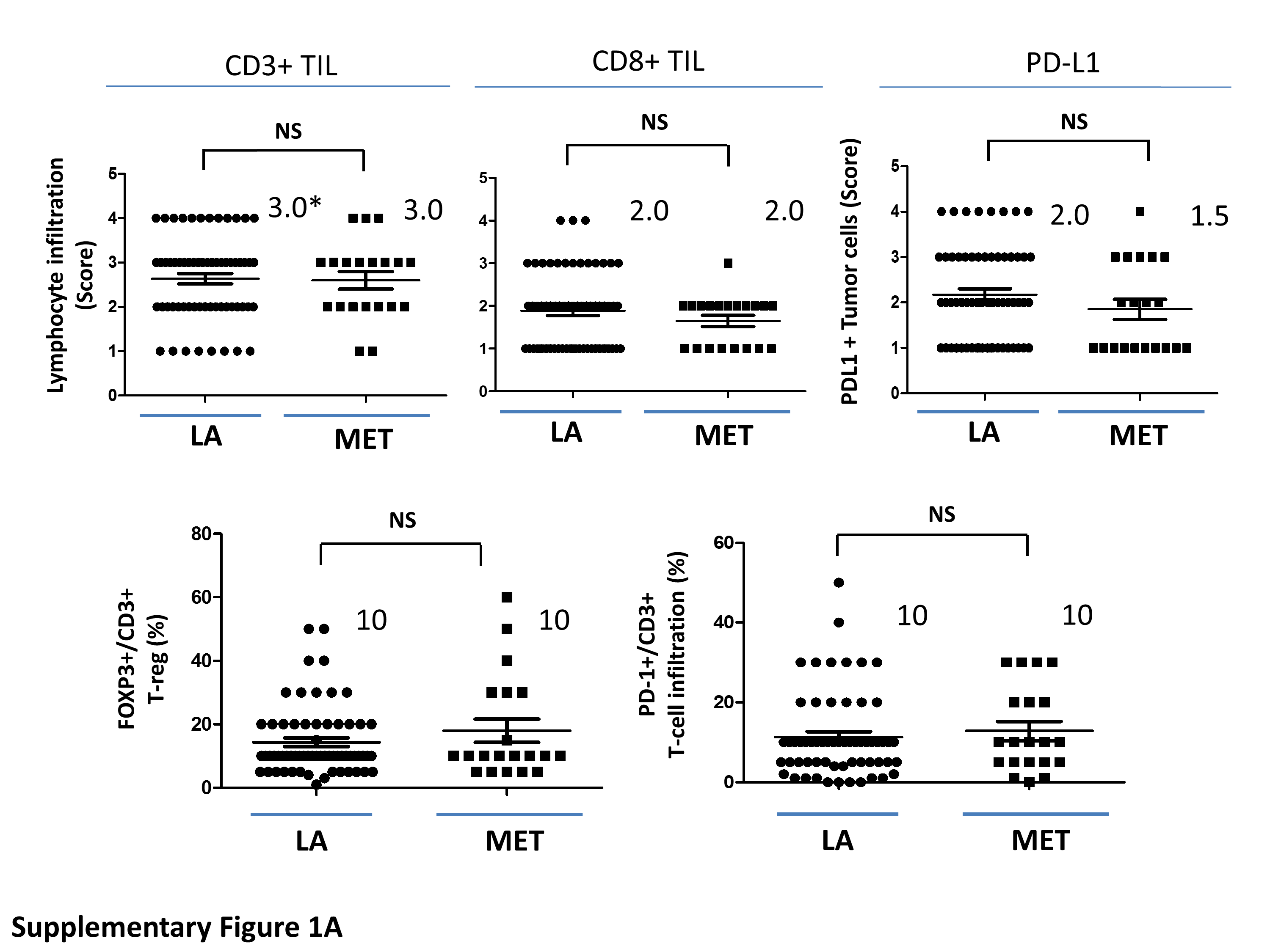

Supplement: Supplementary file 1 — Additional file 1: Supplementary Figure 1. Expression of immune-related markers in LA and MET nasopharyngeal tumors. Difference in CD3+ TIL, CD8+ TIL infiltration, subsets of TIL infiltration and Tumoral PD-L1 expression (membranous or cytoplasmic) between (A) locally advanced (LA, n = 63) versus metastatic (MET, n = 20) nasopharyngeal carcinoma cases or (B) primary tumor (1ry) and metastatic sites (MT sites) in available paired tissues from some of the LA patients before and after relapse and development of metastasis (n = 6). * Numbers indicate the median. [file 12885_2020_6757_MOESM1_ESM.zip › Supplementary Figure 1AR4.TIF]

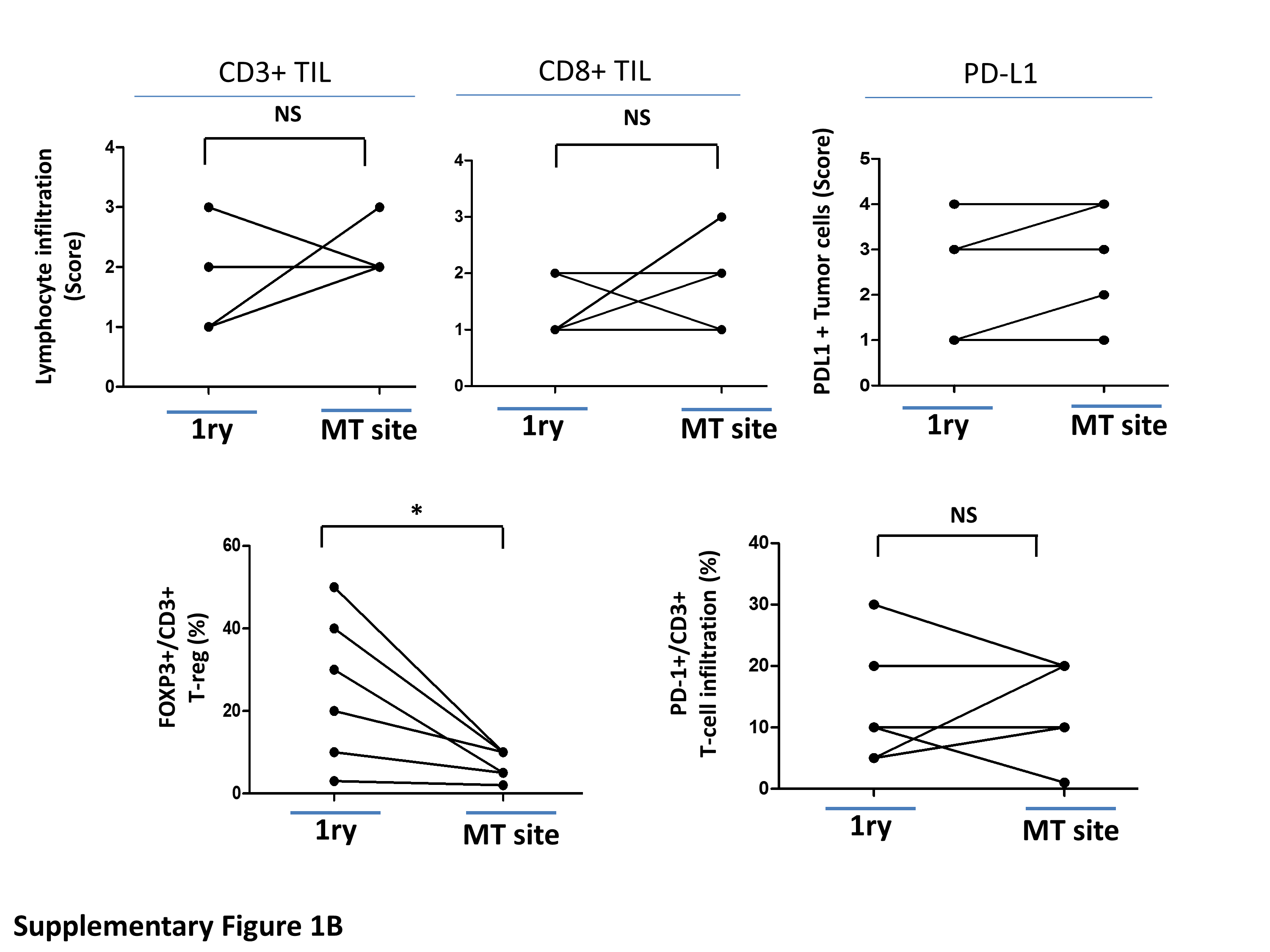

Supplement: Supplementary file 1 — Additional file 1: Supplementary Figure 1. Expression of immune-related markers in LA and MET nasopharyngeal tumors. Difference in CD3+ TIL, CD8+ TIL infiltration, subsets of TIL infiltration and Tumoral PD-L1 expression (membranous or cytoplasmic) between (A) locally advanced (LA, n = 63) versus metastatic (MET, n = 20) nasopharyngeal carcinoma cases or (B) primary tumor (1ry) and metastatic sites (MT sites) in available paired tissues from some of the LA patients before and after relapse and development of metastasis (n = 6). * Numbers indicate the median. [file 12885_2020_6757_MOESM1_ESM.zip › Supplementary Figure 1BR4.TIF]

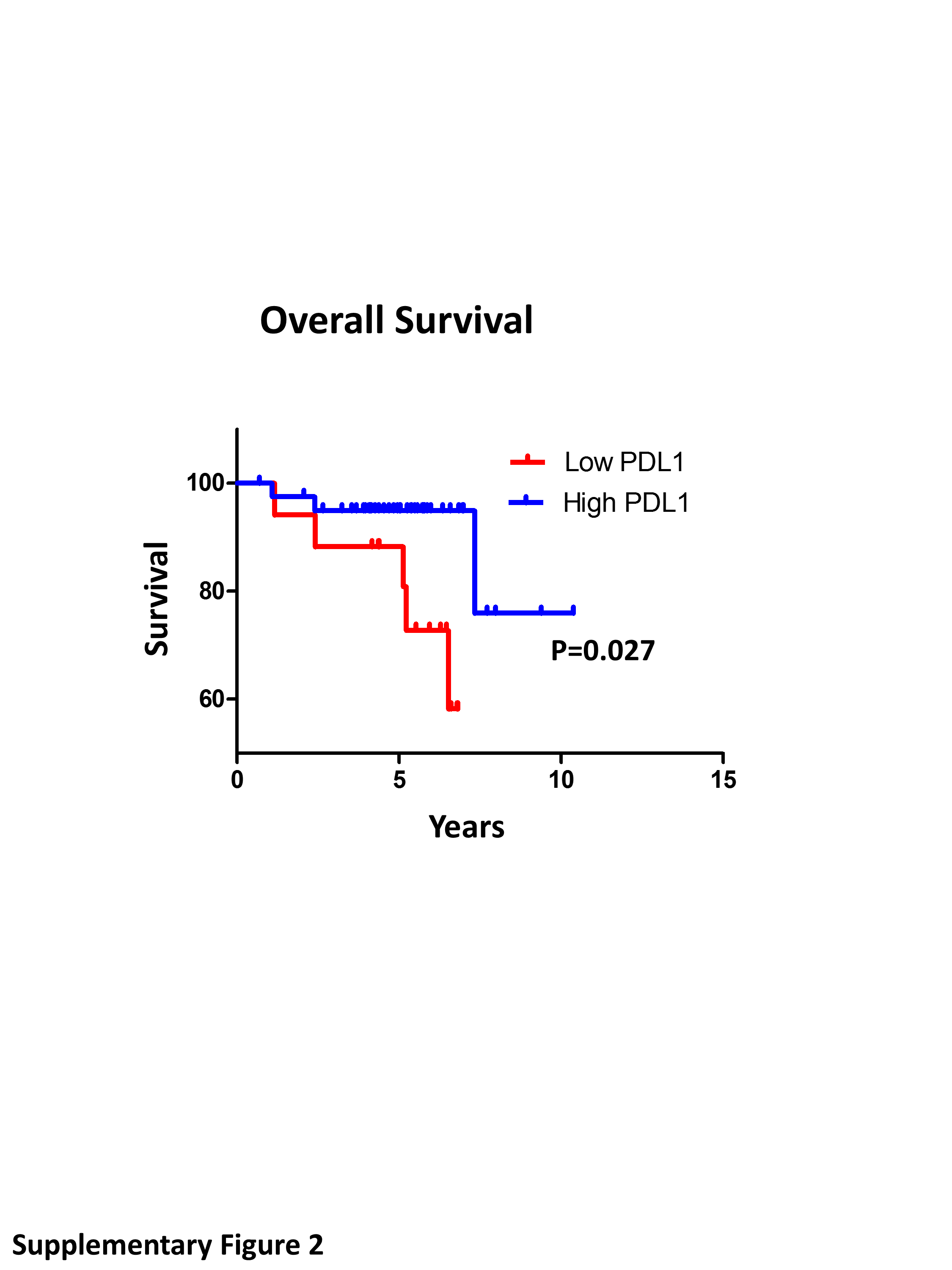

Supplement: Supplementary file 2 — Additional file 2: Supplementary Figure 2. Relation of tumoral PD-L1 expression to survival of WHO type III LA-NPC patients (n = 58). Kaplan–Meier survival curves showing overall survival (OS) of WHO type III LA-NPC patients, in relation to their tumoral PD-L1 expression. Statistical significance was calculated using log-rank test. [file 12885_2020_6757_MOESM2_ESM.tif]

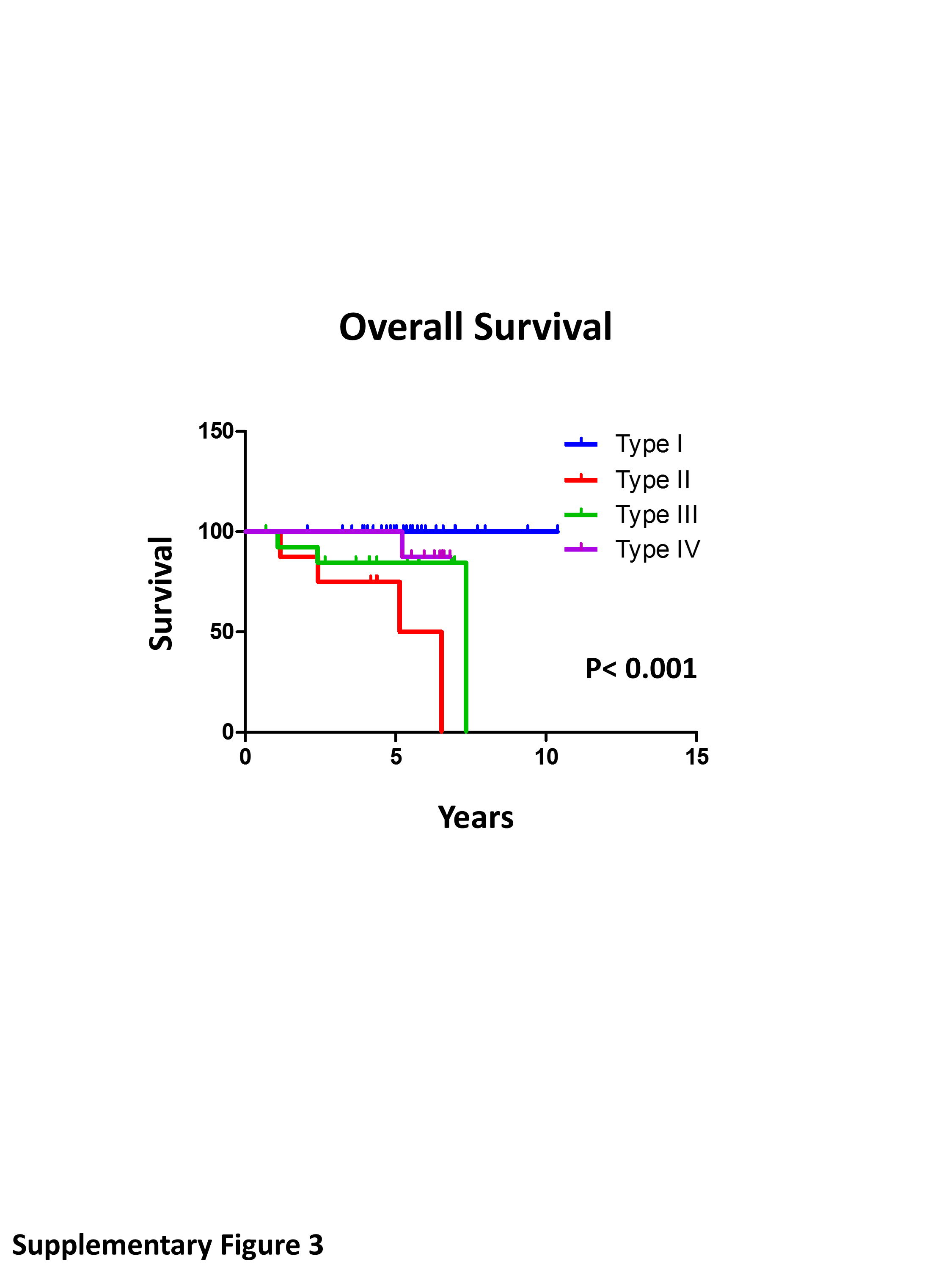

Supplement: Supplementary file 3 — Additional file 3: Supplementary Figure 3. Correlation of microenvironment type with survival of LA-NPC patients. Kaplan–Meier survival curves showing OS of WHO type III LA-NPC patients (n = 58) in relation to their microenvironment type based on CD3+ TIL and PD-L1 expression. Type I: Tumoral PD-L1 is positive and CD3 TIL is high, Type II: PD-L1 is negative while CD3 is low, Type III: tumoral PD-L1 is positive while CD3 TIL is low and Type IV: PD-L1 is negative while CD3 TIL is high. Statistical significance was calculated using log-rank test. [file 12885_2020_6757_MOESM3_ESM.tif]
