# Supplementary material for: CD3+T-lymphocyte infiltration is an independent prognostic factor for advanced nasopharyngeal carcinoma
Source: BMC Cancer. 2020 Mar 21;20:240. doi: 10.1186/s12885-020-06757-w (PMC7227256; doi:10.1186/s12885-020-06757-w)
Supplement: Supplementary file 4 — Additional file 4: Supplementary Table 1. List of Ventana antibodies used in the study. [file 12885_2020_6757_MOESM4_ESM.docx]

**Supplementary Table 1** List of Ventana antibody used in the study.

| **Antibody** | **Clone** | **CAT#** |
| --- | --- | --- |
| **PD-L1** | **SP263** | **790-4905** |
| **CD3** | **2GV6** | **790-4341** |
| **CD8** | **SP57** | **790-4460** |
| **PD-1** | **NAT105** | **760-4895** |
