# Supplementary material for: CD3+T-lymphocyte infiltration is an independent prognostic factor for advanced nasopharyngeal carcinoma
Source: BMC Cancer. 2020 Mar 21;20:240. doi: 10.1186/s12885-020-06757-w (PMC7227256; doi:10.1186/s12885-020-06757-w)
Supplement: Supplementary file 5 — Additional file 5: Supplementary Table 2. Agreement between the two scoring pathologists. [file 12885_2020_6757_MOESM5_ESM.docx]

**Supplementary Table 2** Agreement between the two scoring pathologists

|  |  |
| --- | --- |
|  | **Cohen’s κ *** |
| **║** |  |
| Total TiL | 0.58 (0.38-0.78) |
| CD3+ TIL | 0.61 (0.41-0.81) |
| CD8+ TIL | 0.57 (0.34-79) |

**Abbreviations**: **κ value range from 0 to 1, where 0 indicates perfect disagreement and 1 indicating perfect agreement between pathologists. The strength of the agreement is defined as poor if κ <0, slight if κ was within 0-0.2, fair if κ was within 0.21-0.4, moderate if κ was within 0.41-0.60, substantial if κ is within 0.61-80 and perfect if κ is between 0.81 and 1.0.*
