# Supplementary material for: CD3+T-lymphocyte infiltration is an independent prognostic factor for advanced nasopharyngeal carcinoma
Source: BMC Cancer. 2020 Mar 21;20:240. doi: 10.1186/s12885-020-06757-w (PMC7227256; doi:10.1186/s12885-020-06757-w)
Supplement: Supplementary file 6 — Additional file 6: Supplementary Table 3 Multivariate Cox proportional hazard analysis of clinicopathological features and immunological markers with disease-free survival (DFS) and overall survival (OS) in 58 patients with WHO type III LA-NPC at the time of presentation. [file 12885_2020_6757_MOESM6_ESM.docx]

**Supplementary Table 3** Multivariate Cox proportional hazard analysis of clinicopathological features and immunological markers with disease-free survival (DFS) and overall survival (OS) in 58 patients with WHO type III LA-NPC at the time of presentation.

|  | **DFS** | | |  | **OS** | | |
| --- | --- | --- | --- | --- | --- | --- | --- |
|  | **HR** | **95% CI** | ****P*** |  | **HR** | **95% CI** | ***P*** |
| Age |  |  |  |  |  |  |  |
| < 40 years | 1 |  |  |  | 1 |  |  |
| ≥ 40 years | 0.7 | 0.2-3.1 | 0.678 |  | 0.3 | 0.0-13.6 | 0.525 |
|  |  |  |  |  |  |  |  |
| UICC Stage |  |  |  |  |  |  |  |
| III | 1 |  |  |  | 1 |  |  |
| IV | 0.7 | 0.2-2.8 | 0.621 |  | 0.1 | 0.0-2.6 | 0.184 |
|  |  |  |  |  |  |  |  |
| **CD3+ TIL** |  |  |  |  |  |  |  |
| **High** | **1** |  |  |  | **1** |  |  |
| **Low** | **7.3** | **2.2-28.4** | **<0.001** |  | **243** | **7.0-156540.9** | **<0.001** |
|  |  |  |  |  |  |  |  |
| FOXP3 |  |  |  |  |  |  |  |
| Low (≤ 10% of CD3+TIL) | 1 |  |  |  | 1 |  |  |
| High (>10% of CD3+TIL) | 1.2 | 0.4-3.7 | 0.781 |  | 1.3 | 0.1-38.0 | 0.848 |
|  |  |  |  |  |  |  |  |
| PD-1 |  |  |  |  |  |  |  |
| Low (≤ 10% of CD3+TIL) | **1** |  |  |  | **1** |  |  |
| High (>10% of CD3+TIL) | 0.5 | 0.1-1.5 | 0.202 |  | **0.03** | **0-0.5** | **0.011** |
|  |  |  |  |  |  |  |  |
| PD-L1 |  |  |  |  |  |  |  |
| Positive (≥ 10%) | 1 |  |  |  | **1** |  |  |
| Negative (< 10%) | 1.9 | 0.5-6.8 | 0.318 |  | **136** | **5.3-80852.0** | **<0.001** |

**P* values in bold represent significant data.
