# Supplementary material for: CD3+T-lymphocyte infiltration is an independent prognostic factor for advanced nasopharyngeal carcinoma
Source: BMC Cancer. 2020 Mar 21;20:240. doi: 10.1186/s12885-020-06757-w (PMC7227256; doi:10.1186/s12885-020-06757-w)
Supplement: Supplementary file 7 — Additional file 7: Supplementary Table 4. Univariate Cox proportional hazard regression analysis of different CD3 + TIL/Tumor PD-L1 expression combination types with overall survival (OS) in 58 patients with local NPC at time of presentation. [file 12885_2020_6757_MOESM7_ESM.docx]

**Supplementary Table 4** Univariate Cox proportional hazard regression analysis of CD3+TIL/Tumor PD-L1 expression types with overall survival (OS) in 58 patients with local NPC at time of presentation

|  | **Death** | |  |  | **OS** | | |
| --- | --- | --- | --- | --- | --- | --- | --- |
|  | **-** | **+** |  |  | ***HR*** | ***95% CI*** | ***P**** |
| **║Type of Microenvironment** |  |  |  |  |  |  |  |
| I | **28 (100)^♣^** | **0 (0)** |  |  | **1** |  |  |
| II | **5 (50)** | **5 (50)** |  | II vs I | **8.2 E+9** | **4.9-E+136** | **<0.001** |
| III | **13 (76)** | **4 (24)** |  | III vs I | **1.5 E+9** | **3.1-3.2 E+225** | **0.005** |
| IV | **7 (88)** | **1 (12)** |  | IV vs I | 8.7 E+8 | 0.7- E+36 | 0.070 |

**Abbreviations**: **P* values in bold represent significant data. (+ and -) are number of positive and negative patients, **║** Type of microenvironment: Type I: PD-L1+/CD3+, Type II: PD-L1^neg^/CD3^neg^, Type III: PD-L1+/CD3^neg^, Type4: PD-L1^neg^, CD3+, ^♣^Numbers between brackets are the percentages of patient.
